# BRCA1/2 Reversion Mutations and Cancer Therapy Resistance

**DOI:** 10.3390/biology15110866

**Published:** 2026-05-31

**Authors:** Wenjing Qi, Gege Yang, Yingyi Zhang, Liping Han, Kevin H. Mayo, Xianlu Zeng, Jingang Mo

**Affiliations:** 1 Department of Bioscience, Changchun Normal University, Changchun 130032, China; 2 Biochemistry, Molecular Biology, and Biophysics, Health Sciences Center, University of Minnesota, Minneapolis, MN 55455, USA; 3 Key Laboratory of Molecular Epigenetics of Ministry of Education, College of Life Sciences, Northeast Normal University, Changchun 130024, China

**Keywords:** BRCA1/2, homologous recombination deficient tumors, reversion mutation, chemoresistance

## Abstract

Germline loss-of-function mutations in *BRCA1* and *BRCA2* markedly increase susceptibility to breast, ovarian, and other cancers. Mechanistically, BRCA2 facilitates RAD51 recruitment to sites of DNA damage, whereas BRCA1 regulates homologous recombination repair (HRR) through double-strand break resection and broader DNA damage response signaling. These insights underpin targeted therapies such as poly (ADP-ribose) polymerase inhibitors (PARPis), which induce synthetic lethality in homologous recombination-deficient tumors. Clinically, PARPis have demonstrated significant benefit in BRCA1/2-mutated breast, ovarian, pancreatic, and prostate cancers. However, resistance remains a major obstacle, with secondary intragenic *BRCA1/2* mutations restoring partial protein function representing a prominent mechanism. Despite therapeutic advances, critical gaps persist in understanding how specific BRCA1/2 domains and residual protein activities contribute to tumorigenesis and treatment response. In this review, we summarize the structural and functional domains of BRCA1/2, their pathogenic mutation profiles, and therapeutic strategies targeting BRCA1/2-deficient cancers. Despite therapeutic advances, critical gaps persist in understanding how specific BRCA1/2 domains and residual protein activities contribute to tumorigenesis and treatment response. This review emphasizes the need for functional studies of BRCA1/2 variants to refine risk prediction and develop mutation-tailored therapies.

## Simple Summary

Inherited mutations in the *BRCA1* and *BRCA2* genes greatly increase a person’s chances of developing breast, ovarian, and other cancers. These genes normally produce proteins that repair damaged DNA. The BRCA2 protein helps guide a key repair protein called RAD51 to broken DNA, while BRCA1 manages the initial processing of DNA breaks and coordinates repair signals. Drugs known as PARP inhibitors take advantage of this repair weakness to destroy cancer cells, and they have successfully treated BRCA-mutated cancers of the breast, ovary, pancreas, and prostate. Unfortunately, tumors often become resistant when additional mutations partially restore the BRCA protein’s ability to work. A major challenge is that scientists still do not fully understand how different regions of the BRCA proteins influence cancer risk and treatment outcomes. This review summarizes the structure and function of BRCA1 and BRCA2, the profiles of harmful mutations, and current therapeutic approaches. The authors stress that detailed functional studies of individual mutations are essential. Such research will sharpen cancer risk predictions for people carrying these mutations and enable the design of mutation-specific therapies, ultimately overcoming resistance and improving patient care.

## 1. Introduction

In the 1990s, inherited loss-of-function variants in *BRCA1* or *BRCA2* were first identified as major drivers of familial predisposition to early-onset breast and ovarian cancers [1,2,3]. Over the past 30 years, extensive research has deepened our understanding of the biological functions of these tumor suppressor genes. Heterozygous germline mutations in either *BRCA1* or *BRCA2* that compromise normal protein function markedly increase the risk of breast, ovarian, and several other malignancies [4,5]. Although multiple mechanisms have been proposed to explain the tissue-specific tumorigenic consequences of *BRCA1* and *BRCA2* dysfunction, their fundamental role in safeguarding genome integrity has emerged as the prevailing paradigm.

BRCA1 and BRCA2 are indispensable mediators of homologous recombination repair (HRR), a critical pathway for resolving DNA lesions such as double-strand breaks (DSBs) and replication fork collapse [6]. In the canonical HRR pathway, BRCA2 directly binds to the recombinase RAD51 and promotes its recruitment to sites of DNA damage. Although RAD51 recruitment is also impaired in BRCA1-deficient cells, BRCA1 exerts broader functions within the DNA damage response, including the regulation of DSB end resection and the orchestration of HRR-related signaling cascades [7]. Additional activities of BRCA1, including chromatin remodeling [8] and transcriptional regulation [9], are also thought to contribute to its tumor suppressor function. These mechanistic insights have provided the foundation for developing targeted therapeutic strategies against BRCA1/2-mutated cancers [10]. Among these strategies, poly (ADP-ribose) polymerase inhibitors (PARPis) represent one of the most significant breakthroughs. Based on the concept of synthetic lethality, PARPis selectively eliminate HR-deficient tumor cells while sparing normal tissues. Clinically, four PARPis have been approved for the treatment of breast, ovarian, pancreatic, and prostate cancers carrying pathogenic *BRCA1/2* mutations, and they are used in neoadjuvant, adjuvant, and maintenance settings [11,12].

However, resistance to PARPis has emerged as a major therapeutic barrier. Initially, secondary intragenic mutations in *BRCA2* were identified as drivers of resistance to both PARPis and platinum agents [13,14]. Subsequent clinical studies revealed that somatic reversion mutations in *BRCA1*, *BRCA2*, *RAD51C*, *RAD51D*, or *PALB2* can restore HRR activity, thereby conferring resistance to PARPis and platinum-based chemotherapy across multiple tumor types, including ovarian, breast, pancreatic, and prostate cancers [14,15,16,17,18,19,20,21]. Despite these insights, our understanding of BRCA1/2 as tumor suppressors remains limited. Elucidating the specific roles of BRCA1/2 functional domains is critical not only for advancing cancer prevention strategies but also for designing mutation-informed therapeutic interventions. In this review, we first provide a critical evaluation of the structural and functional domains of BRCA1/2, their pathogenic mutation spectra, and current therapeutic strategies for BRCA1/2-mutated cancers. We then examine the role of reversion mutations in therapy resistance and discuss emerging approaches to overcome BRCA-associated resistance mechanisms.

## 2. Literature Search Strategy

This narrative review was based on peer-reviewed literature retrieved from PubMed, Web of Science, and Scopus, with supplementary records from ScienceDirect. The primary search period covered January 2020 to May 2026, although several seminal studies published since 1994 were included to provide foundational and mechanistic context. Search terms included combinations of “BRCA1/2,” “cancer therapy,” “pathogenic mutations,” “reversion mutations,” “hypomorphic BRCA isoforms,” “PARP inhibitors,” and “cancer therapy resistance” using Boolean operators (AND/OR). Reference lists of relevant articles were also manually screened. Priority was given to original research articles, genomic and transcriptomic studies, meta-analyses, and authoritative reviews published in English-language SCI-indexed journals. Conference abstracts, non-peer-reviewed reports, and studies lacking methodological rigor were excluded. As this was a narrative review, no formal quality assessment, risk-of-bias analysis, or PRISMA-guided screening was performed; studies were selected based on relevance, scientific rigor, and contribution to the field.

## 3. Overview of BRCA1/2

### 3.1. BRCA1/2 Structure and Function

**Figure 1:**
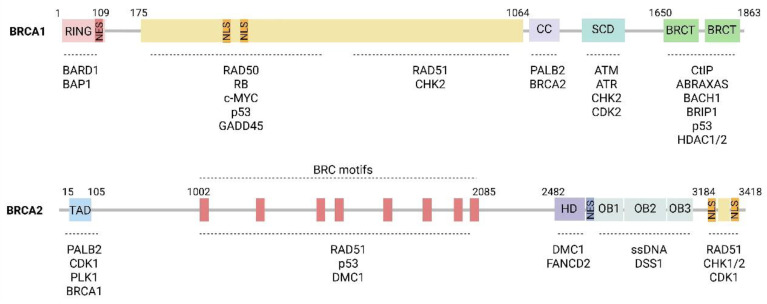
Functional domains of BRCA1/2 and their interacting partners. BRCA1 comprises a N-terminal RING domain (interacting with BARD1, BAP1), a central region with NLS (with RAD50, RAD51, RB, c-MYC, GADD45, p53, CHK2), a coiled-coil (CC) domain (with ALB2, BRCA2), a serine cluster domain (SCD; with ATM, ATR, CHK1/2), and a C-terminal BRCT domain (with BACH1, ABRAXAS, CtIP, BRIP, p53, HDAC1/2). BRCA2 contains a PALB2-interacting TAD (with CDK1, PLK1, BRCA1), eight BRC repeats (with RAD51, p53, DMC1), a DNA-binding domain (DBD; HD + three OB folds; binds to DMC1, FANCD2, ssDNA), an NLS with CDK1 phosphorylation sites, and a C-terminal domain required for RAD51 binding.

*BRCA1* and *BRCA2* function as classical tumor suppressor genes. Although both play essential roles in DNA repair, their structures and molecular functions exhibit distinct characteristics. The *BRCA1* gene spans approximately 81 kb on chromosome 17q21.31 and consists of 24 exons, encoding a protein of 1863 amino acids [22,23]. Structurally, BRCA1 contains three major domains: the N-terminal Really Interesting New Gene (RING) domain, a large central region encoded by exons 11–13, and the C-terminal BRCA1 C-Terminus (BRCT) domain (Figure 1). The RING domain harbors a zinc-binding RING finger motif, which confers E3 ubiquitin ligase activity through heterodimerization with BRCA1-associated RING domain protein 1 (BARD1). This interaction also masks the C-terminal nuclear export sequences (NES) within both proteins, thereby ensuring nuclear retention of the BRCA1–BARD1 complex [24]. The central region, which accounts for ~65% of the protein, contains multiple interaction sites for DNA repair proteins such as RAD50, RAD51, retinoblastoma protein (Rb), and c-Myc [24,25]. This region also harbors two nuclear localization sequences (NLS), a coiled-coil domain that mediates binding to partner and localizer of BRCA2 (PALB2), and a serine cluster domain (SCD). The C-terminal region contains tandem BRCT repeats, which function as phospho-serine binding modules that recognize serine-X-X-phenylalanine (SXXF) motifs in DNA damage response proteins, including C-terminal binding protein 1-interacting protein (CtIP), BRCA1 A complex subunit (ABRAXAS), BRCA1-interacting protein C-terminal helicase 1 (BRIP1), BTB and CNC homology 1 (BACH1), and p53 [23,24]. Pathogenic mutations in BRCA1 are distributed throughout the entire coding region of the protein, indicating that multiple functional domains contribute to its tumor suppressor activity [26]. However, whether all its functions are required to confer resistance to platinum drugs or PARPi remains controversial. BRCA1 hypomorphic variants lacking part or all of the N-terminal RING domain have been reported to promote resistance to both PARPi and platinum agents [27,28]. Likewise, alternative splicing isoforms of *BRCA1* (BRCA1 ∆11 or ∆11q) have also been implicated in therapeutic resistance [29]. In addition, hypomorphic BRCA1 proteins lacking one or both BRCT domains have been associated with chaperone-mediated stabilization and genomic rearrangements, thereby contributing to treatment resistance [30].

The *BRCA2* gene spans approximately 84 kb on chromosome 13q13.1 and comprises 27 exons, encoding a protein of 3418 amino acids. Unlike BRCA1, BRCA2 lacks intrinsic enzymatic activity and instead functions as a scaffold protein organized into three main regions: the N-terminal transcriptional activation domain (TAD), a central region with eight BRC repeats, and a C-terminal DNA-binding domain (DBD) (Figure 1) [23]. The N-terminal TAD mediates interaction with PALB2 and includes phosphorylation sites for cyclin-dependent kinase 1 (CDK1) at Ser93, Thr64, and Thr77, as well as for polo-like kinase 1 (PLK1) at Ser193, Ser205, Ser206, Thr203, and Thr207 [31,32]. Exon 11, the largest exon, encodes eight highly conserved BRC repeats (~35 amino acids each) that bind monomeric RAD51 and regulate its recruitment to DNA damage sites. The C-terminal region comprises a DBD with one helical domain and three oligonucleotide-binding (OB) folds, enabling BRCA2 to interact with both single- and double-stranded DNA [32,33]. This region also contains three NLS and a C-terminal RAD51 interaction domain (CTRD), which stabilizes the BRCA2–RAD51 nucleoprotein filament. Phosphorylation sites for CDKs and checkpoint kinases CHK1/2 are also present in this region (e.g., Ser3284, Ser3291, Ser3319, Thr3310, Thr3323, and Ser3387) [31]. Although pathogenic mutations are distributed at relatively similar frequencies throughout BRCA2, reversion mutations occur unevenly across the gene. Clinically, reversions are more frequently observed within the N-terminal “hotspot” region (c.750–775), whereas they are comparatively rare in the C-terminal “cold spot” region beyond c.7617 [15]. BRCA2 is widely recognized for its critical role in HRR through interaction with RAD51. Notably, BRCA2 contains two distinct RAD51-binding regions: the central BRC repeat motifs and a C-terminal RAD51-binding domain [34]. Reversion mutations affecting these domains may partially or fully restore HRR activity, thereby contributing to resistance to platinum-based chemotherapy and PARPis.

Although early studies suggested that BRCA1 might function as a transcription factor [35,36], subsequent findings established that both BRCA1 and BRCA2 colocalize with RAD51 in nuclear foci, underscoring their roles in maintaining genome stability [37]. Functional studies in Brca1- or Brca2-deficient mouse embryonic fibroblasts revealed spontaneous chromosomal abnormalities and impaired HRR, confirming their pivotal role in maintaining genome integrity [38,39,40,41]. Both proteins also participate in activating and maintaining the G2/M checkpoint, thereby preventing the propagation of DNA damage into daughter cells [38,42,43,44,45]. Importantly, BRCA2 is indispensable for RAD51 function. Cells lacking the BRCA2 C-terminal RAD51-binding domain show hypersensitivity to γ-irradiation but retain intact G1–S and G2–M checkpoints, indicating that BRCA2 deficiency primarily compromises RAD51-mediated HR rather than cell cycle regulation [46]. Further studies demonstrated that BRCA2 governs RAD51 localization and DNA binding, and its loss diminishes HR activity following DNA double-strand breaks [47,48,49]. These findings firmly establish BRCA1 and BRCA2 as central guardians of genome integrity through HR-mediated repair, providing the foundation for subsequent advances in understanding DNA repair mechanisms and their links to tumorigenesis.

### 3.2. Pathogenic Mutations of BRCA1/2 in Cancer

Germline mutations in *BRCA1* or *BRCA2* markedly increase lifetime cancer risk, with carriers facing up to an 85% risk of breast cancer and a 15–56% risk of ovarian cancer [50]. Loss of BRCA1/2 function leads to homologous recombination deficiency (HRD) and accumulation of DSBs, which are thought to be major drivers of tumorigenesis [40]. BRCA1/2 also maintain genome stability by protecting stalled replication forks [51,52], preventing the accumulation of single-stranded DNA gaps [53,54,55], and suppressing R-loops formed by RNA–DNA hybrids [56,57,58]. Defects in these additional functions may also contribute to genomic instability in cancer. Besides germline or somatic loss-of-function mutations, BRCA1/2 expression can be silenced through promoter hypermethylation and other epigenetic mechanisms [59,60]. Despite functional similarities, BRCA1- and BRCA2-mutated tumors exhibit distinct clinical and molecular characteristics. *BRCA1* mutation carriers are predisposed to triple-negative breast cancer (TNBC), which typically presents at an early age and carries a high relapse risk. By contrast, *BRCA2* mutation carriers more often develop luminal-like breast cancers that are generally responsive to antiestrogen therapy [47,61,62]. Pathogenic variants in *BRCA1/2* are continuously catalogued in publicly available databases such as the Breast Cancer Information Core (BIC), BRCA Exchange, and ClinVar (https://brcaexchange.org/factsheet; https://www.ncbi.nlm.nih.gov/clinvar/, accessed on 1 January 2020). Table 1 summarizes the most common BRCA1 and BRCA2 mutations registered in the databases. Approximately 7% of variants listed in BRCA Exchange are pathogenic, with three founder mutations—185delAG (*BRCA1*), 5382insC (*BRCA1*), and 6174delT (*BRCA2*)—being among the most common [22].

Cancer risk associated with *BRCA1/2* mutations is influenced by their genomic location, leading to the identification of breast cancer cluster regions (BCCRs) and ovarian cancer cluster regions (OCCRs). In *BRCA1*, two BCCRs are located in the N- and C-terminal regions (BCCR1: c.179–505; BCCR2: c.4328–4945; BCCR2′: c.5261–5563), encompassing the RING and BRCT domains, respectively. The OCCR is located in exon 11 (c.1380–4062). In *BRCA2*, BCCRs map to the N-terminal region (BCCR1: c.1–596; BCCR1′: c.772–1806) and the DNA-binding domain (BCCR2: c.7394–8904). OCCRs are located in the central region, including exon 11 within the BRC repeats (OCCR1: c.3249–5681; c.5946, OCCR2: c.6645–7471). Notably, the 6174delT founder mutation lies within OCCR1, conferring a relatively higher risk of ovarian than breast cancer [64]. Interestingly, male carriers of OCCR1 variants in *BRCA2*, including 6174delT, exhibit reduced prostate cancer (PCa) risk compared with carriers of non-OCCR mutations. By contrast, a distinct prostate cancer cluster region (PCCR) has been mapped to c.7914–3′ of *BRCA2*, with no equivalent identified in *BRCA1* (Table 1) [65,66,67,68].

*BRCA1/2* mutations are also clinically relevant in pancreatic cancer (PC). Pancreatic ductal adenocarcinoma (PDAC), which accounts for ~90% of pancreatic cancers, has a poor prognosis with a 5-year survival rate below 9% [69]. Approximately 10% of PDACs are hereditary [70]. Early studies estimated the prevalence of *BRCA1/2* germline mutations in PDAC at 4–5% [71], but the POLO trial later reported an incidence of 6–7% in metastatic PDAC [72]. In high-risk populations, such as Ashkenazi Jews—where the 185delAG, 5382insC (*BRCA1*), and 6174delT (*BRCA2*) mutations are common—prevalence can reach 20% [73,74]. Current data indicate *BRCA1* mutations in 2.4% and *BRCA2* mutations in 5.7% of PDAC cases, with PDAC ranking as the third most common cancer in germline *BRCA2* carriers [63,75,76,77].

The concept of “BRCAness” has been proposed to describe tumors with HRD caused by BRCA1/2 loss or defects in other HR genes [78]. This phenotype not only explains the genomic instability driving tumorigenesis but also provides a therapeutic framework by highlighting specific vulnerabilities that can be exploited in BRCA1/2-mutant cancers.

### 3.3. Treatment of BRCA1/2 Mutated Cancers

**Figure 2:**
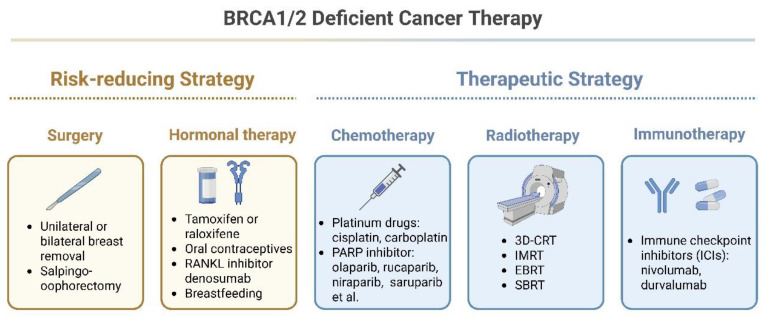
Therapeutic Strategies for BRCA1/2 Deficient Cancers. Overview of current treatment and risk-reduction approaches for BRCA1/2-associated cancers, including risk-reducing surgery (mastectomy, salpingo-oophorectomy), hormonal therapy (tamoxifen, denosumab), chemotherapy (platinum agents, PARP inhibitors), radiotherapy (3D-CRT, IMRT, EBRT, SBRT), and immunotherapy with immune checkpoint inhibitors (e.g., nivolumab, durvalumab).

*BRCA1* and *BRCA2* are frequently mutated in breast, ovarian, prostate, pancreatic, and other cancers. A variety of preventive and therapeutic strategies have been developed to mitigate cancer risk and improve outcomes in mutation carriers (Figure 2).

#### 3.3.1. Risk-Reducing Strategies

To lower the elevated lifetime risk in *BRCA1/2* mutation carriers, risk-reducing surgeries are widely implemented. Prophylactic mastectomy, involving unilateral or bilateral breast removal, reduces breast cancer risk by approximately 90% [79]. Similarly, risk-reducing salpingo-oophorectomy (RRSO), which removes the ovaries and fallopian tubes, decreases ovarian cancer risk by up to 96% [80]. However, these surgeries have a massive impact on the quality of life. RRSO induces premature “surgical menopause,” associated with fatigue, anxiety, cardiovascular dysfunction, and sleep disturbance [81,82,83]. Hormone replacement therapy is frequently recommended to alleviate these symptoms [84,85,86]. For individuals declining surgery, alternative strategies remain limited. Regular screening with mammography and magnetic resonance imaging (MRI) is standard, while chemo-preventive approaches such as selective estrogen receptor modulators or aromatase inhibitors have been proposed but lack large-scale validation [87]. Epidemiological studies suggest that breastfeeding may lower cancer risk in BRCA1/2 carriers, potentially via tissue differentiation and reduced estrogen exposure [88]. Similarly, oral contraceptives appear to lower ovarian cancer risk, possibly through pro-apoptotic effects on ovarian and fallopian tube epithelia [89,90]. More recently, inhibition of the RANKL/RANK pathway has emerged as a preventive approach. Preclinical studies demonstrated delayed mammary tumor onset, and biopsies from patients treated with the RANKL inhibitor denosumab showed reduced proliferation. A clinical trial (NCT04711109) is underway to evaluate denosumab as a preventive option for *BRCA1* carriers [91,92]. While surgical interventions remain the most effective preventive strategies, ongoing research seeks less invasive alternatives to preserve quality of life.

#### 3.3.2. Platinum-Based Chemotherapies

BRCA1/2-deficient tumors are highly sensitive to interstrand crosslink (ICL)-inducing agents, particularly platinum compounds such as cisplatin and carboplatin [78,93,94]. Approved since 1978 for multiple malignancies [95], platinum drugs generate DNA adducts and cross-links that exploit HR deficiency. Preclinical models of BRCA1-deficient breast cancer demonstrated pronounced cisplatin sensitivity [96]. These findings align with a recent clinical study in TNBC patients, which reported that carboplatin had double the objective response rate of docetaxel in BRCA1/2 mutation carriers, whereas there was no difference in an unselected population [97].

#### 3.3.3. PARP Inhibition and Synthetic Lethality

Despite the initial sensitivity of BRCA1/2 mutated tumors to platinum drugs, their efficacy is hindered by dose-limiting toxicity in normal tissues and the frequent development of resistance [98,99]. Secondary intragenic BRCA2 mutations restoring partial protein function have been identified as a mechanism of platinum resistance [13,14]. These insights paved the way for exploiting synthetic lethality with PARP inhibition. Two landmark studies demonstrated that poly (ADP-ribose) polymerase 1 (PARP1) inhibitors selectively kill BRCA1/2-deficient cells [100,101]. PARP1 detects single-strand breaks (SSBs) and double-strand breaks (DSBs), undergoes auto-PARylation, and recruits downstream repair proteins [102]. PARPis not only block repair but also trap PARP1 on DNA, generating cytotoxic DNA–protein complexes that collapse replication forks and induce DSBs [103]. In HR-deficient cells, these lesions are repaired by error-prone mechanisms, enhancing tumor-specific lethality. Additional studies show PARPi-induced replication gaps [6,104] and transcription–replication conflicts, further exacerbating DNA damage in HR-deficient cells [105]. These discoveries led to the approval of four PARPis, olaparib, rucaparib, niraparib, and talazoparib in the United States and Europe, and two PARPis, fuzuloparib and pamiparib in China, for patients with BRCA1/2-mutated cancers [106,107].

Currently approved PARPis are nonselective, targeting both PARP1 and PARP2 [108,109]. To improve therapeutic specificity, saruparib (AZD5305), a second-generation PARP1-selective inhibitor and potent DNA trapper, was recently developed. Preclinical studies demonstrated robust activity in BRCA2-mutant patient-derived xenograft (PDX) models, both as monotherapy and in combination with carboplatin [47,108,110,111,112]. These results suggest that selective inhibition of PARP1 may preserve efficacy while minimizing toxicity.

#### 3.3.4. Immunomodulatory Strategies

Beyond DNA repair inhibition, PARPis exert immunomodulatory effects. Olaparib induces cytosolic DNA fragments that activate the cGAS–STING pathway, leading to dendritic cell activation, CD8^+^ T-cell infiltration, and type I interferon production [113]. Cyclic GMP–AMP synthase (cGAS) recognizes cytosolic DNA and generates cGAMP, which activates the adaptor STING and its downstream immune pathway [114]. This cascade enhances tumor immunogenicity, upregulates chemokines (e.g., CCL5, CXCL10), and increases PD-L1 expression [115,116]. However, PARPis can also upregulate PD-L1 expression primarily via GSK3β inactivation, indicating that PARP inhibitors render cancer cells more resistant against T-cell-mediated cell death [117,118]. These findings provide a rationale for combining PARPis with immune checkpoint inhibitors (ICIs) [119]. Clinical trials are now testing PARPi–ICI combinations, including anti-CTLA4 and anti-PD-L1 agents [120,121,122,123]. In the phase II CheckMate 9KD trial, metastatic castration-resistant prostate cancer (mCRPC) patients received olaparib with nivolumab. While overall results were modest in unselected patients, improved responses and survival outcomes were observed in homologous recombination repair–positive cohorts [124,125]. Together, these strategies highlight a rapidly evolving therapeutic landscape for BRCA1/2-mutated cancers. Ongoing efforts using genetically engineered models and humanized PDX systems will further elucidate synthetic lethal interactions and guide the design of more effective, durable treatment strategies.

## 4. BRCA1/2 Reversion Mutations in Therapy Resistance

As discussed in the previous section, although platinum-based chemotherapies and PARPis have significantly improved the management of BRCA1/2-mutated cancers, their long-term efficacy is hampered by the inevitable development of resistance. Clinically, only about half of patients with BRCA1/2 mutations initially respond to PARPi treatment [126], and the majority eventually acquire resistance during therapy [60]. Likewise, resistance to platinum agents is frequently observed in ovarian and breast cancers, where relapse occurs despite favorable initial responses [13]. This adaptive capacity of tumor cells poses a major barrier to durable therapeutic success.

Resistance to PARPis can emerge through multiple mechanisms. Tumor cells may upregulate DNA damage repair factors such as RAD51, thereby partially restoring homologous recombination activity; increase drug efflux via multidrug resistance transporters such as multidrug resistance gene 1 (MDR1); or express hypomorphic isoforms of BRCA proteins that retain partial function [63]. These adaptations attenuate the synthetic lethal effects of PARP inhibition. Notably, platinum agents—long used in the treatment of ovarian carcinoma, including in patients with BRCA1/2 mutations—likely share overlapping mechanisms of action and resistance with PARPis [13].

**Figure 3:**
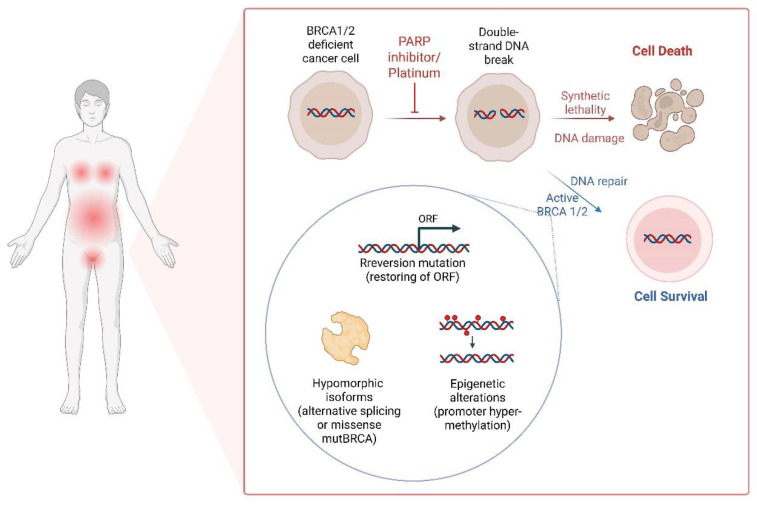
BRCA1/2 reversion mutation promotes cancer chemoresistance. In BRCA1/2-deficient cells, loss of homologous recombination (HR) together with PARP inhibition induces genomic instability and cell death. Reversion or secondary mutations can restore the *BRCA1/2* open reading frame, re-establishing HR. Additionally, epigenetic alterations and hypomorphic isoforms may partially restore BRCA1/2 function, thereby promoting cell survival and resistance to therapy.

Among these resistance pathways, BRCA1/2reversion mutations represent the most clinically significant and widely documented mechanism. Such secondary mutations restore the open reading frame of the mutant allele, enabling re-expression of a functional BRCA protein and thereby reinstating homologous recombination proficiency [13,14]. Multiple studies have identified these reversion events in tumor samples from patients who progressed on PARPi therapy, and importantly, their presence has also been linked to cross-resistance to platinum-based chemotherapy [16,21,127]. Moreover, certain hypomorphic *BRCA1* alleles confer partial DNA repair capacity, further driving resistance to both PARPis and platinum drugs (Figure 3) [27,29,47,128,129]. In this section, we focus on the spectrum of BRCA1/2 reversion mutations and their pivotal role in mediating chemoresistance (Table 2).

### 4.1. Reversion Mutations Restoring the Open Reading Frame (ORF)

Platinum-based chemotherapy and PARPis exert their cytotoxicity by inducing DNA damage-mediated cell death. Loss-of-function mutations in BRCA1/2 impair homologous recombination repair (HRR), sensitizing tumors to these agents. However, secondary alterations in *BRCA1/2* can restore the open reading frame (ORF), re-establish partial or full protein function, and consequently drive resistance to both platinum drugs and PARPis [13,16,20,135]. Such reversion events arise through point mutations, small insertions/deletions, or large genomic rearrangements that circumvent the original deleterious mutation (Figure 3) [14,15,16,136,137,138].

A large Japanese pan-cancer study (*n* = 3738 patients, 32 cancer types) identified somatic *BRCA* reversion mutations in tumor tissue or circulating cell-free DNA (cfDNA). Among 208 patients harboring pathogenic *BRCA1/2* variants, 21 reversion mutations were detected in 12 individuals (3 in *BRCA1*, 18 in *BRCA2*) [130]. One notable case involved breast cancer, where a reversion in BRCA2 p.L24*—within the PALB2-binding domain—was detected. In BRCA2, exon 11 encodes the BRC repeats and RAD51-binding domains essential for HRR. Reversions in this region frequently involve long deletions (up to 2493 bp) that span multiple BRC repeats, restore the ORF, and confer resistance to platinum therapy [130]. Interestingly, complete loss of all BRC repeats has not been reported, suggesting that at least two repeats are indispensable for BRCA2’s functional activity and resistance acquisition [139]. A study in CAPAN1 pancreatic cancer, which carries the protein-truncating c.6174delT frameshift mutation showed that new BRCA2 isoforms were expressed in the resistant lines as a result of intragenic deletion of the c.6174delT mutation and restoration of the ORF [13]. Reversions have also been observed in gallbladder cancer, a noncanonical BRCA-associated malignancy, underscoring the broader relevance of this mechanism [130].

Splice-site mutations can also lead to reversion through alternative splicing. For example, deletions affecting *BRCA2* intron 7, exon 8, intron 8, exon 9, and part of intron 9 were shown to generate splice variants that bypassed a pathogenic exon 9 acceptor mutation. In another patient carrying a *BRCA2* c.7008-1G>A splice acceptor mutation in exon 14, multiple independent secondary alterations were identified, including three single-nucleotide variants (c.7010C>G, c.7013C>G, c.7016A>G) and two distinct 8 bp deletions in exon 13. These secondary mutations, all present in cis with the primary pathogenic mutation but not co-occurring within the same sequencing read, indicate that they arose as independent events. Notably, because the secondary mutations were positioned at 3 bp intervals adjacent to an adenine, each could plausibly reconstitute an ‘AG’ consensus splice acceptor site, thereby restoring correct exon 13–14 splicing and the open reading frame of *BRCA2* [21].

In prostate cancer, *BRCA2* reversion mutations have been linked to resistance against PARPis such as olaparib and talazoparib. One reported case involved a germline nonsense mutation in *BRCA2* (p.K1872X, chr13:32,914,106 A→T), which introduces a premature stop codon in exon 11 and truncates the protein at residue 1872. Following initial sensitivity to talazoparib, resistant biopsies revealed two secondary *BRCA2* alleles with deletions of 177 bp and 66 bp, respectively. Both deletions removed the pathogenic p.K1872X site, restored the open reading frame, and preserved the essential C-terminal domain of BRCA2. The resulting proteins, predicted to be 3359 and 3396 amino acids in length, carried alterations in the BRC repeat domain 7 but retained sufficient HRR capacity to confer drug resistance [13]. Similar reversion deletions affecting BRC repeats 5–8 have been shown in cell line models to produce BRCA2 proteins with partial HRR activity, sufficient to drive resistance to both platinum agents and PARPis. A second case described a germline heterozygous two-nucleotide deletion in *BRCA2* (p.R259fs), which introduced a premature stop codon at residue 274. Resistant tumor samples carried 105 somatic alterations upstream of the lesion, most clustered in exons 9–10. Thirty-four distinct indels were predicted to restore the ORF, and indels in exon 9 were confirmed to occur in cis with p.R259fs, consistent with a tumor retaining only a single *BRCA2* allele [17].

In addition, sequencing of pretreated tumor biopsies has revealed large *BRCA1* deletion events that restore the ORF in patients with platinum-refractory cancers. In a patient with platinum-resistant cancer harboring a somatic *BRCA1* mutation (c.1045G>T; p.E349*), cfDNA analysis identified four distinct reversion mutations, including base substitutions and deletions restoring the ORF. Another patient with a somatic *BRCA1* mutation (c.2679delG; p.K894fs) exhibited eight unique reversion mutations in cfDNA, consisting of deletions ranging from 2 to 29 bp [16]. Collectively, these clinical observations underscore the plasticity of BRCA1/2 in acquiring diverse secondary mutations that restore ORF integrity and reconstitute HRR, thereby enabling tumors to escape the synthetic lethality induced by PARPi and platinum therapies.

### 4.2. Hypomorphic BRCA1/2

A clinical study among patients with high-grade serous ovarian cancer (HGSOC) harboring BRCA1 alterations, showed that those with frameshift mutations within exon 11 exhibit poorer survival and reduced responses to platinum-based therapy compared with patients carrying frameshift mutations outside this region [29,140]. This difference may be attributed to therapy resistance arising from the overexpression of BRCA1 splice isoforms lacking most or all of exon 11 (sometimes referred to as exon 10, but herein designated exon 11) [29]. The BRCA1 Δ11q isoform, generated by alternative splicing within exon 11 (c.788–4096), produces a truncated but partially functional protein. A second isoform, Δ11, which skips the entire exon 11 (c.671–4096), is less abundant in human tumor cell lines [132]. Nonetheless, evidence from mice model suggests that Δ11 can partially compensate for the loss of full-length BRCA1, particularly in a *TP53*-deficient background [141]. Importantly, both Δ11 and Δ11q transcripts exclude the exon containing pathogenic variants and thereby evade nonsense-mediated decay (NMD). Although hypomorphic, these isoforms retain sufficient HRR capacity to confer resistance to PARPis and platinum drugs in tumors with *BRCA1* exon 11 mutations (Figure 3) [29]. Recent studies using nine patient-derived xenograft (PDX) models of HGSOC, TNBC and ovarian carcinosarcoma, supported by cell line and genomic analyses, found that two of five PARPi-resistant PDXs harbored secondary BRCA1 splice-site mutations (SSMs). These SSMs promoted alternative splicing, restored partial BRCA1 activity, and conferred PARPi resistance in both HGSOC and TNBC models. Furthermore, truncated but functional hypomorphic BRCA1 proteins—including the BRCA1-11q splice isoform, RING-deficient BRCA1 generated by downstream translation initiation, and HSP90-stabilized C-terminal mutants [131]—were detected by immunofluorescence in resistant PDXs. These isoforms retained the ability to form nuclear foci and were associated with both primary and acquired PARPi resistance [142]. Despite these insights, the clinical relevance and molecular mechanisms underlying *BRCA1* exon skipping remain incompletely understood.

### 4.3. Epigenetic Alterations

Epigenetic plasticity also contributes to therapeutic resistance. In sporadic TNBC PDX models with BRCA1 mutations, *BRCA1* promoter hypermethylation is a common mechanism of gene silencing [133]. Resistance can emerge through promoter demethylation, which restores *BRCA1* transcription and HR activity, thereby conferring resistance to PARPis (Figure 3) [133]. Interestingly, a prior study reported that *BRCA1* could remain transcriptionally active under the control of a heterogeneous alternative promoter driven by chromosomal rearrangement, even in the presence of promoter hypermethylation [119,133]. In ovarian cancer, clinical data indicate that loss of *BRCA1* methylation is associated with acquired chemotherapy resistance [60,143]. These findings underscore the plasticity of epigenetic regulation in modulating BRCA1 function and highlight the need to consider epigenetic dynamics in resistance monitoring.

### 4.4. Tumor Microenvironment Alterations

Beyond tumor cell–intrinsic mechanisms, drug resistance is also shaped by the tumor microenvironment (TME). Components of the TME can influence PARPi response through multiple processes, including modulation of intratumoral drug distribution and crosstalk with the immune system. For instance, activation of the stimulator of interferon genes (STING) pathway within tumors has been shown to enhance the recruitment of CD8^+^ T cells, thereby shaping sensitivity to PARPis in BRCA-deficient models [11,113]. An analysis of all reported clinical reversions showed that compensatory frameshift reversions of *BRCA1/2* that fail to restore the original codon (i.e., second-site reversions) generate out-of-frame stretches of novel amino acid sequences absent in the wild-type protein. These aberrant sequences may not be stably expressed but are predicted to encode immunogenic neoantigens, potentially altering the tumor immune microenvironment and contributing to immune evasion [15].

To date, secondary *BRCA* mutations represent the predominant clinically validated resistance mechanism. However, their frequency varies markedly across tumor types, reflecting the heterogeneity of BRCA-mutated cancers and their profound genomic instability [130,144]. For instance, restoration of BRCA function occurs in approximately 15–25% of ovarian cancers progressing under olaparib treatment [16,20], but this frequency rises to nearly 60% in patients with BRCA-mutated metastatic breast cancer [21]. Similarly, genetic reversion of both somatic and germline *BRCA2* mutations has been documented in smaller cohorts of metastatic castration-resistant prostate cancer patients following progression on olaparib [17,18]. Although these studies provide important insights, the generalizability of *BRCA* reversion frequencies remains limited by differences in PARPi agents used across trials, disease-specific biology, and patient stratification criteria. Addressing these limitations will require harmonized clinical and translational approaches to fully capture the spectrum of resistance mechanisms across tumor types.

## 5. Attempts to Overcome BRCA1/2 Resistance

Resistance to therapy in BRCA1/2-mutated cancers may arise through multiple mechanisms, including secondary intragenic mutations, epigenetic reversions that restore the ORF, intrachromosomal rearrangements, or promoter demethylation, all of which can re-establish BRCA function [13,145]. Notably, tumors harboring heterozygous mutant *BRCA* alleles or loss of heterozygosity are often associated with resistance to PARPis and DNA-damaging agents, due to partial or restored HR activity [63,146,147].

### 5.1. Targeting Other DNA Repair Factors

**Figure 4:**
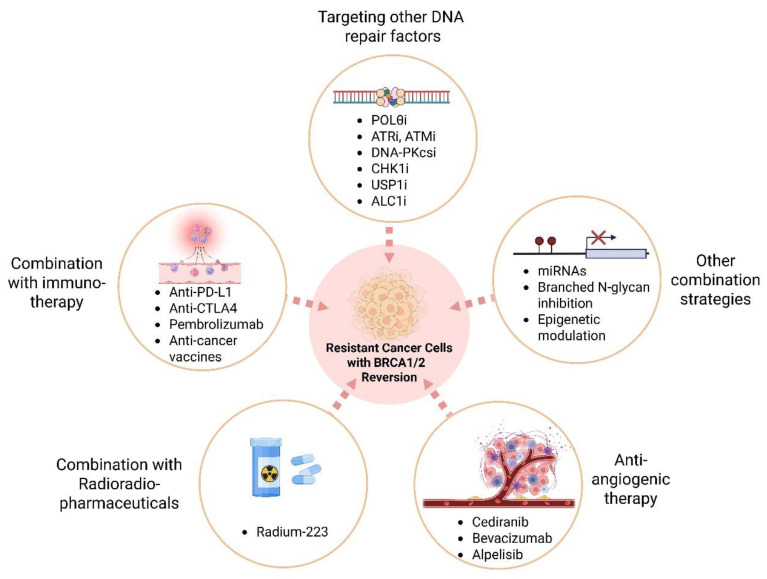
Emerging combination strategies targeting BRCA1/2-resistant cancers. Therapeutic approaches include inhibitors of alternative DNA repair pathways (e.g., POLθ, ATR, ATM, DNA-PKcs, CHK1, USP1, ALC1), combinations with immunotherapy (immune checkpoint blockade, vaccines), targeting reversion-mediated resistance (miRNAs, branched N-glycan inhibition, epigenetic modulators), and other strategies such as radiopharmaceuticals (radium-223) and anti-angiogenic agents (cediranib, bevacizumab).

To extend therapeutic benefit beyond germline *BRCA1/2* mutations, efforts have focused on exploiting HR deficiency in tumors with somatic mutations in DNA repair genes or those exhibiting a “BRCAness” phenotype [63,148]. Inducing “BRCAness” through the use of small-molecule inhibitors targeting DNA repair pathways, in combination with PARPis, represents a promising area of investigation (Figure 4).

One emerging target is DNA polymerase theta (Polθ), encoded by the *POLQ* gene. Polθ is a key mediator of theta-mediated end-joining (TMEJ), a backup repair pathway for resected DSBs when non-homologous end joining (NHEJ) and HR are compromised. Although broadly expressed in normal tissues, Polθ is frequently overexpressed in cancers, correlating with poor prognosis and shorter relapse-free survival. HR-deficient tumors display mutational signatures consistent with error-prone TMEJ, and loss of BRCA1/2 amplifies this dependency, suggesting a synthetic lethal relationship between *POLQ* and HR deficiency. Recent studies also highlight a role for Polθ in filling post-replicative gaps [149,150,151], indicating that both TMEJ inhibition and gap accumulation contribute to synthetic lethality in BRCA-deficient cells [152,153,154,155]. Preclinical studies of Polθ inhibitors (Polθi) in BRCA-deficient models have yielded variable results. While Polθi monotherapy demonstrated low to modest toxicity in different BRCA-deficient cell and organoid models [149,150,154,156], in some cases, supra-physiological concentrations were required to achieve a therapeutic response [11,157]. These findings suggest that Polθ and PARP inhibition may act through distinct mechanisms, positioning Polθ inhibition as a potential therapeutic strategy, particularly in cancers with BRCA reversion-mediated PARPi resistance.

ATM, ATR, and CHK1 are also critical regulators of DNA damage response. In the phase II TRAP trial, patients with mCRPC receiving olaparib plus the ATM inhibitor ceralasertib achieved prostate-specific antigen (PSA) responses of up to 40% in those with HRR or ATM mutations [125], albeit with increased adverse events. Similarly, studies in olaparib-resistant ovarian cancer showed that combining olaparib with ATR or CHK1 inhibitors abrogated compensatory protein upregulation, overcoming PARPi resistance and exerting antiproliferative effects in BRCA2-mutant, olaparib-resistant models [158]. Histone H2AX also contributes to synthetic lethality in BRCA-deficient tumors by facilitating replication fork degradation upon DNA damage. Loss of H2AX restores replication fork stability and induces chemoresistance in BRCA1/2-mutated cells without rescuing HR, suggesting that targeting H2AX-dependent pathways may hold therapeutic potential, particularly in metastatic settings [11,159]. Another promising target is the deubiquitinase USP1, an essential factor in BRCA-mutated and HR-deficient tumors. Combination treatment with a USP1 inhibitor (KSQ-4279) and a PARPi was well tolerated and produced durable tumor regression across several patient-derived PARPi-resistant models [160], supporting the continued clinical development of USP1 inhibitors. Additional approaches include disrupting the RAD51–BRCA2 interaction with small-molecule inhibitors such as dihydroquinolone pyrazoline derivatives (e.g., 35d), which have shown synergistic activity with olaparib in preclinical pancreatic cancer models [161,162]. RAD52 inhibitors also represent a compelling strategy. Although RAD52 functions independently of BRCA2, it is indispensable for RAD51-mediated HR in BRCA2-deficient cells, while playing a negligible role in wild-type cells [163].

Other DNA repair regulators, such as ALC1, further expand the therapeutic landscape. ALC1 enhances apurinic/apyrimidinic endonuclease 1 (APE1) cleavage of abasic sites occluded by nucleosomes. In ALC1-deficient cells, unrepaired abasic sites at replication forks are cleaved by APE1, inducing fork collapse and PARP trapping upon PARPi treatment, thereby driving hypersensitivity [164]. Likewise, deficiency of POLE3–POLE4, accessory subunits of DNA polymerase epsilon (Polε), sensitizes cells to PARPis through PRIMPOL-dependent gap accumulation, bypassing the need for BRCA1 and counteracting common resistance mechanisms [165].

### 5.2. Combination of PARP Inhibitors with Immune Checkpoint Inhibitors

Recent studies have suggested that many BRCA1/2 reversion events generate immunogenic neoantigens, offering potential therapeutic opportunities. Compensatory frameshift reversions that fail to restore the original codon (i.e., second-site reversions) produce out-of-frame peptide sequences of 2–30 amino acids or novel breakpoint junctions absent in the wild-type allele. These unique sequences, often unrecognized by the host immune system, represent potential neoantigens (Figure 4) [15]. Accordingly, tumors harboring such mutations may be amenable to immunotherapeutic approaches, including CAR-T cell therapy, immune checkpoint blockade, or anticancer vaccines. In addition, BRCA1/2 transcriptionally upregulates MGAT5, an enzyme that catalyzes the synthesis of branched N-glycans. In HR-proficient tumors, branched N-glycans enhance resistance to anti–PD-L1 therapy by strengthening PD-L1/PD-1 interactions on CD8^+^ T cells. In orthotopic, syngeneic epithelial ovarian cancer models, inhibition of branched N-glycans using 2-deoxy-D-glucose restored sensitivity to anti–PD-L1 in HR-proficient, but not HR-deficient, tumors [166]. These findings highlight branched N-glycans as promising therapeutic targets and suggest that their inhibition may synergize with PARPis to overcome resistance driven by BRCA1/2 reversion.

### 5.3. Other Combination Strategies

Beyond immunotherapy, several additional combinatorial approaches are being explored to overcome PARPi resistance (Figure 4). In mCRPC, the COMRADE phase I/II trial investigated the safety and efficacy of combining olaparib with the radiopharmaceutical radium-223 in patients with bone metastases. The trial reported a 6-month radiographic progression-free survival (rPFS) of 58%, with the greatest benefit observed in patients harboring homologous recombination repair mutations [125,167].

Epigenetic regulation also plays a role in PARPi response. In serous ovarian cancer, differential expression analyses identified several microRNAs (miRNAs) associated with olaparib resistance (e.g., miR-99b-5p, miR-424-3p, miR-505-5p) and re-sensitization (e.g., miR-324-5p, miR-424-3p). Reconstruction of miRNA–mRNA regulatory networks suggests that these miRNAs may serve as predictive biomarkers for resistance and therapeutic response [168,169]. Epigenetic modifiers involved in transcriptional regulation or chromatin remodelling can enhance PARP-related DNA damage, decrease BRCA1 levels, and increase genomic instability. Preclinical data suggest that inhibiting bromodomains and histone deacetylases (HDACs) can enhance the activity of PARPis [12,170].

Another promising avenue involves targeting tumor angiogenesis, which supports tumor growth, survival, and metastasis. Preclinical studies indicate that anti-angiogenic agents may enhance PARPi efficacy by modulating HRR pathways [171,172]. For instance, cediranib, an anti-angiogenic agent, induces hypoxia and disrupts HRR, thereby sensitizing tumors to PARPis. This effect appears particularly relevant in BRCA1/2 wild-type tumors or those harboring reversion mutations [173,174,175].

Taken together, these findings highlight diverse strategies to overcome BRCA1/2-associated PARPi resistance, spanning DNA repair pathway inhibition, immunotherapy, radiopharmaceuticals, anti-angiogenic agents, and epigenetic regulators. While preclinical and early clinical data are encouraging, unresolved challenges—including treatment-related toxicity and optimal patient stratification—underscore the need for continued refinement of these combination approaches to achieve durable clinical benefit.

## 6. Conclusions

The DNA damage response (DDR) network is indispensable for preserving genomic integrity and enabling cellular survival. As DDR pathways are frequently dysregulated in cancer, they present exploitable therapeutic vulnerabilities. BRCA1 and BRCA2 are pivotal DDR regulators whose loss impairs HR, thereby fueling genomic instability. Despite significant progress, approximately 70% of *BRCA1/2* coding regions remain poorly characterized, highlighting the need for deeper mechanistic studies to delineate how individual domains shape DNA repair and tumorigenesis. The advent of PARPis, built on the principle of synthetic lethality, has transformed the treatment of BRCA1/2-mutated cancers and represents a landmark in precision oncology. Nevertheless, not all patients derive durable benefit, and resistance—most prominently via BRCA1/2 reversion mutations restoring HR function—poses a major obstacle. Dissecting the molecular determinants of these reversion events will be essential for informing rational drug combinations and designing next-generation therapeutic strategies.

### Future Directions

Future research should expand the use of physiologically relevant models, including patient-derived organoids and longitudinal tumor biopsies, to better capture the dynamics of drug sensitivity and acquired resistance. In parallel, increasing attention should be directed toward the roles of tumor heterogeneity and the TME in shaping therapeutic responses. The TME, composed of stromal cells, immune components, and soluble mediators, not only promotes tumor progression and metastasis but also contributes substantially to therapeutic resistance. Emerging evidence suggests that modulation of the TME may restore drug sensitivity and improve treatment efficacy. In addition, the integration of longitudinal molecular monitoring strategies, such as circulating cfDNA analysis, methylation profiling, repeat tumor biopsies, immunogenic neoepitope detection, and serial sequencing, may facilitate the early identification of reversion mutations and guide personalized clinical intervention. Collectively, these advances could improve the durability of therapeutic responses and ultimately enhance clinical outcomes for patients with BRCA1/2-mutated cancers.

## Figures and Tables

**Table 1:** Mutations of BRCA1/2 and Functional Consequences.

Gene	Mutation Site/Type	Domain	Cancer Cluster Regions	Functional Consequence
*BRCA1*	C24 *, C64 *, E23fs (185delAG), C61 *	RING	BCCR1: c.179–505	Disrupt interaction with BARD1, BAP1; affect ubiquitin ligase activity
	R163 *, N277 *, N319fs, N363fs, S551 *, V627 *, Y655 *, S694 *, S753 *, I815fs, P832fs, R951 *, I1019 *, N1121 *, S1178 *, Y1202 *, S1218 *	Central region	OCCR: c.1380–4062 (exon 11)	Alter p53, RAD50, GADD45, c-MYC, CHK2, and RAD51 interaction
	Y1429 *, D1533fs, S1613 *	Serine cluster domain (SCD)	-	Affect ATM/ATR/CHK2-dependent phosphorylation, DNA damage response
	R1670fs, E1682 *, Y1845 *, Q1756fs (5382insC)	C-terminal	BCCR2: c.4328–4945; BCCR2′: c.5261–5563	Disrupt interactions with transcriptional regulators (p53, HDAC1/2, CtIP, etc.)
*BRCA2*	Y42 *, D153 *, S205 *, D244 *, S309 *, I332fs, L398fs	TAD	BCCR1: c.1–596; BCCR1′: c.772–1806	Disrupt interaction with PALB2, BRCA1, RAD51 loading
	C315fs (6174delT), Y1069 *, T1150fs, L1686 *, E1734 *, S1741 *, Y1751 *, E1806 *, K1875 *, S1989 *	BRC repeats	OCCR1: c.3249–5681, c.5946 delT; OCCR2: c.6645–7471	Alter RAD51 binding, homologous recombination
	E2328 *, S2509 *, I2490 *	C-terminal (HD domain)	-	Impair DMC1/FANCD2 interaction
	E2641 *, E2663 *, G2793 *, Y3049 *, L3061 *, K3326 *	C-terminal (DBD/OB fold)	BCCR2: c.7394–8904 PCCR: c.7914–3′	Impair ssDNA binding

Notes: BRCA mutations are according to the Breast cancer Information Core (BIC), the BRCA Exchange, and ClinVar [22,63,64,65]. fs, frame shift. *, nonsense mutations.

**Table 2:** BRCA1/2 Secondary Alterations Driving Resistance.

Cancer Type	Primary Mutation	Secondary Mutation/Alteration	Resistance Mechanism	
Breast cancer	BRCA2 Y1655 * exon11 (c.4965C>A)	C1654-T1656del (c.4959-4967del) Y1655L (c.4964-4965AC>TA) K1649-T1656del (c.4947_4970del24) T1653-T1656del (c.4958-4969delCTTGTTACACAA)	Restore the open reading frame (ORF); retain RAD51-binding capacity	[130]
	BRCA2 L24 * exon3 (c.71T>G)	L24W(c.71-72TA>GG)	Frameshift to restore the ORF	[130]
	BRCA2 I605 fs exon10 (c.1813del)	T598-G602>KNTER (c.1793-1806CATCTTATAAAGGA>AAAATACCGAAAGG)	Frameshift to restore the ORF	[130]
	BRCA2 exon 9–14 splice acceptor mutations (e.g., c.7008-1G>A)	Alternative splicing via secondary SNVs (c.7010C>G, c.7013C>G, c.7016A>G in exon14) and deletions (two 8 bp deletions in exon13)	Reconstitute functional splice acceptor sites; restore ORF	[21]
	BRCA1 E1214 * exon 11 (c.3640G>T)	K1183-T1256>RG (c.3548-3767>GAGG)	Restore ORF	[130]
Pancreatic cancer	BRCA2 c.6174delT (exon11 26050-26052)	Intragenic deletion: exon 11-exon27 (25451-83997; 25375-83990), exon11-exon22 (25312-65211), exon11 (25857-26316)	Restore ORF, capable of nuclear translocation and RAD51 interaction	[13]
	BRCA2 I605 fs exon10 (c.1813del)	I605_Q609>YRKTK (c.1813_1825ATACCGAAAGAAA>TACCGAAAGACCA)	Restore ORF	[130]
	BRCA2 L904fs exon11 (c.5709del)	L1908FS (c.5722-5723del)	Frameshift	[130]
Prostate cancer	BRCA2 A2854fs exon20 (c.8589_8590insA)	A2864S (c.8590_8591GC>AG) A2864T(c.8590G>A) A2864_T2867>SLIH (c.8590-8601GCCTTATTAACT>AGCCTTATTAAC)	Re-establish mutation-induced disruption of the ORF	[130]
	BRCA2 K1872X (nonsense, exon 11)	Secondary deletions (177 bp and 66 bp) removing the pathogenic K1872X mutation	Restore ORF; preserve C-terminal domain; partial homologous recombination repair (HRR) restored	[17]
	BRCA2 R259fs (frameshift)	>100 somatic indels clustered in exons 9-10; 34 predicted to restore ORF	Re-express functional BRCA2	[17]
Ovarian cancer	BRCA2 c.6174delT (exon11 26050-26052)	Intragenic deletion: exon11 (26058-26197; 26056-26165)	Restore ORF	[13]
	BRCA2 R2318 * exon13 (c.6952C>T)	R2318W (c.6952-6954CGA>TGG)	Mutation to restore ORF and HRR activity	[130]
	BRCA2 Y1655 * exon11 (c.4965C>A)	Y1655F (c.4964A>T)	Mutation to restore ORF and HRR activity	
	BRCA1 frameshift mutations within exon 11	Alternative splicing BRCA1 Δ11q (partial skipping of exon 11), Δ11 isoforms (skipping of exon 11)	Hypomorphic isoforms evade nonsense-mediated mRNA decay (NMD); retain partial HRR	[29,131,132]
	BRCA1 E1214 * exon 11 (c.3640G>T)	E1214W (c.3640-3641GA>TG)	Restore ORF	[130]
	BRCA1 E349 * (c.1045G>T) (nonsense); BRCA1 K894fs (c.2679delG)	Multiple distinct reversion mutations detected in cfDNA (base substitutions and small deletions) E349G (c.1046A>G), E349D (c.1047G>T), L347_P359del (c.1039_1077del39), D345_K351del (c.1035-1055del21), G914fs (c.2740-2750delGAGTCTAATAT)	Restore ORF; reconstitute HRR	[16]
Gallbladder cancer	BRCA2 E881 * exon 11 (c.2641G>T)	Long deletions spanning BRC repeats: S879-D885del (c.2635-2655del21), D878-S1121del (c.2633_3364del732), E653-T1483del (c.1959-4451del2493) E881Y (c.2641-2643GAA>TAT)	Restore ORF and HRR activity	[130]
Ovarian & breast cancer	BRCA1 promoter hypermethylation (silencing)	Loss of methylation; BRCA1 de novo gene fusions (T127cis1 and T127nim2)	Promoter demethylation; gene rearrangements, leading to a heterologous promoter control; restore HRR	[133,134]

Note: *, nonsense mutations.

## Data Availability

No new data were created or analyzed in this study. Data sharing is not applicable.

## Abbreviations

HRR	homologous recombination repair
PARPis	poly (ADP-ribose) polymerase inhibitors
DSBs	double-strand breaks
RING	interesting new gene
BRCT	BRCA1 C-terminus
BARD1	BRCA1-associated RING domain protein 1
NES	nuclear export sequences
Rb	retinoblastoma protein
NLS	nuclear localization sequence
PALB2	partner and localizer of BRCA2
SCD	serine cluster domain
CtIP	C-terminal binding protein 1-interacting protein
ABRAXAS	BRCA1 A complex subunit
BRIP1	BRCA1-interacting protein C-terminal helicase 1
BACH1	BTB and CNC homology 1
TAD	transcriptional activation domain
DBD	DNA-binding domain
CDK1	cyclin-dependent kinase 1
PLK1	polo-like kinase 1
HRD	homologous recombination deficiency
TNBC	triple-negative breast cancer
BIC	Breast Cancer Information Core
PCa	prostate cancer
PC	pancreatic cancer
PDAC	pancreatic ductal adenocarcinoma
RRSO	risk-reducing salpingo-oophorectomy
SSBs	single-strand breaks
PARP1	poly (ADP-ribose) polymerase 1
PDX	patient-derived xenograft
cGAS	cyclic GMP–AMP synthase
ICIs	immune checkpoint inhibitors
mCRPC	metastatic castration-resistant prostate cancer
MDR1	multidrug resistance gene 1
ORF	open reading frame
HGSOC	high-grade serous ovarian cancer
NMD	nonsense-mediated decay
SSMs	splice-site mutations
TME	tumor microenvironment
Polθ	DNA polymerase theta
TMEJ	theta-mediated end-joining
NHEJ	non-homologous end joining
PSA	prostate-specific antigen
miRNAs	microRNAs
